# One-year results of cemented bipolar radial head prostheses for comminuted radial head fractures

**DOI:** 10.3205/iprs000071

**Published:** 2015-12-15

**Authors:** Reinhold Laun, Michael Wild, Mohssen Hakimi

**Affiliations:** 1 Vivantes Klinikum Neukölln, Department of Orthopedic and Trauma Surgery, Berlin, Germany; 2 Klinikum Darmstadt, Department of Orthopedic, Trauma and Hand Surgery, Darmstadt, Germany; 3 Vivantes Klinikum Am Urban, Department of Orthopedic, Trauma and Hand Surgery, Berlin, Germany

**Keywords:** radial head fracture, bipolar radial head prosthesis, treatment elbow injuries

## Abstract

**Introduction: **Comminuted radial head fractures (Mason type III) continue to pose a challenge to orthopedic surgeons. When internal fixation is not possible, radial head arthroplasty has been advocated as the treatment of choice. The purpose of this retrospective study was to evaluate clinical and radiological short-term results of patients with Mason type III radial head fractures treated with a cemented bipolar radial prosthesis.

**Methods: **Twelve patients received cemented bipolar radial head hemiarthroplasty for comminuted radial head fractures. In all patients a CT scan was obtained prior to surgical treatment to assess all associated injuries. Postoperatively an early motion protocol was applied. All patients were evaluated clinically and radiologically at an average of 12.7 months.

**Results: **According to the Mayo Modified Wrist Score, the Mayo Elbow Performance Score, the functional rating index of Broberg and Morrey, and the DASH Score good to excellent results were obtained. Grip strength and range of motion were almost at the level of the unaffected contralateral side. Patient satisfaction was high, no instability or signs of loosening of the implant, and only mild signs of osteoarthritis were seen.

**Conclusion: **Overall good to excellent short-term results for primary arthroplasty for comminuted radial head fractures were observed. These encouraging results warrant the conduction of further studies with long-term follow-up and more cases to see if these short-term results can be maintained over time.

## Zusammenfassung

**Einleitung: **Radiuskopftrümmerfrakturen (Mason Typ III) stellen weiterhin eine Herausforderung für den Unfallchirurgen und Orthopäden dar. Wenn eine osteosynthetische Versorgung nicht möglich ist, repräsentiert die Implantation einer Radiuskopfprothese die Behandlung der Wahl. Das Ziel dieser retrospektiven Studie war die Evaluation klinischer und radiologischer Ein-Jahres-Ergebnisse von Patienten, die nach einer Mason Typ III Fraktur mit einer zementierten bipolaren Radiuskopfprothese behandelt worden waren.

**Methoden: **Zwölf Patienten erhielten eine zementierte bipolare Radiuskopfprothese nach einer Radiuskopftrümmerfraktur. Bei allen Patienten wurde präoperativ eine Computertomographie zur Erfassung aller Begleitverletzungen durchgeführt. Postoperativ wurde ein frühfunktionelles Nachbehandlungsschema angewandt. Alle Patienten konnten durchschnittlich nach 12.7 Monaten klinisch und radiologisch nachuntersucht werden.

**Ergebnisse: **Entsprechend des Mayo Modified Wrist Scores, des Mayo Elbow Performance Scores, des Functional Rating Index von Broberg and Morrey und des DASH Scores konnten gute bis exzellente Ergebnisse erzielt werden. Griffstärke und Bewegungsausmaß zeigten sich auf dem Niveau der unverletzten Gegenseite. Die Patientenzufriedenheit war durchweg hoch. Es zeigten sich keine persistierenden Instabilitäten oder Lockerungszeichen der Prothesen. Es wurden lediglich geringe Anzeichen einer Arthrose nachgewiesen.

**Schlussfolgerung: **Insgesamt konnten gute bis exzellente Ein-Jahres-Ergebnisse bei der primären Behandlung von Radiuskopftrümmerfrakturen mittels Radiuskopfprothese nachgewiesen werden. Diese vielversprechenden Ergebnisse sollten mittels weiterer Studien mit größeren Patientenzahlen und längerem Nachbeobachtungszeitraum weiter evaluiert werden.

## Introduction

Radial head fractures are frequent injuries accounting for 33% of all elbow fractures [1]. However, comminuted radial head fractures (Mason Type III) where reconstruction is not feasible continue to pose a challenge to orthopedic surgeons. These unstable fractures of the radial head commonly occur as part of a complex injury pattern and are associated with other osseous and ligamentous injuries to the elbow or forearm [2] [3]. Treatment with open reduction and internal fixation has resulted in a high rate of complications [1]. In cases where internal fixation is not possible, surgical management includes radial head excision with or without radial head replacement [4]. Radial head excision alone has resulted in complications, such as valgus elbow instability, elbow stiffness, and proximal migration of the radius and therefore fallen out of favor [1]. The radial head represents an important stabilizer of the elbow joint especially in case of injuries to the collateral ligaments and the interosseous membrane [5]. Therefore, radial head arthroplasty has been advocated for the treatment of unreconstructable radial head fractures with concomitant medial collateral ligament injury, interosseous ligament injury, or elbow dislocation [1]. It is assumed that radial head replacement leads to comparable kinematics, stability, and load transfer to an intact elbow [4]. Furthermore, implantation of a radial head prosthesis restores the height of the lateral column and ensures both elbow stability in the coronal plane and vertical stability of the forearm [6]. A variety of radial head prostheses are available. Implants differ with respect to the design of their head and stem, as well as, materials and mechanisms of fixation. Furthermore they can be divided into monopolar or bipolar concepts [4].

Although radial head prostheses have an important role in the treatment of complex radial head fractures, little data exist on the outcome for different design concepts. The purpose of this retrospective study was to evaluate clinical and radiological short-term results of patients with Mason Type III radial head fractures treated with a cemented bipolar radial prosthesis.

## Materials and methods

**Figure 1:**
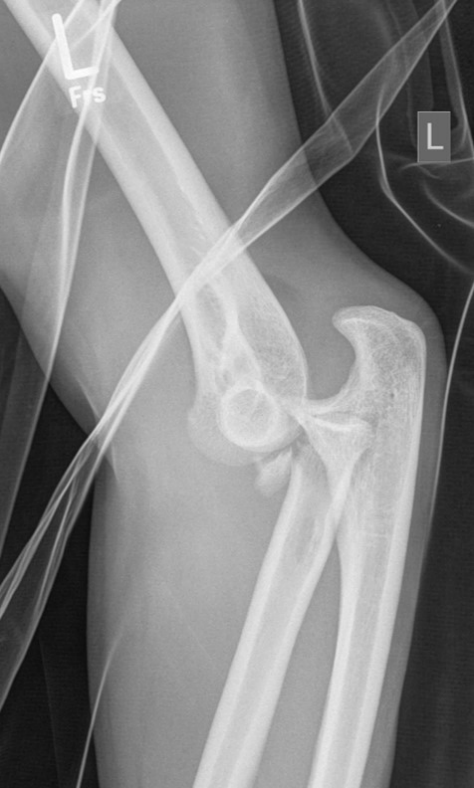
Female patient (36 years) with a Mason type III fracture of the radial head combined with a posterior elbow dislocation. Radiographs on the day of the injury.

**Figure 2:**
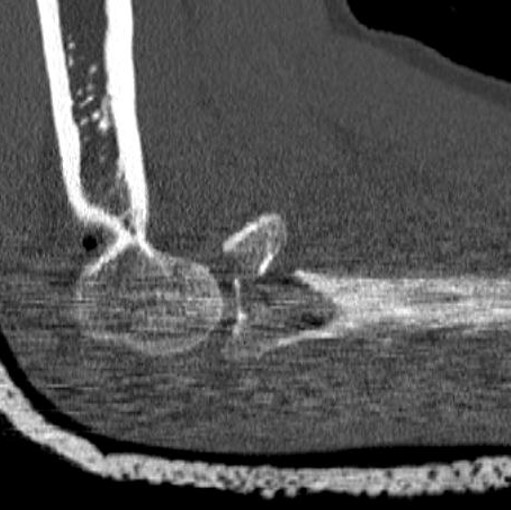
Same patient: preoperative CT scans, demonstrating the Mason Type III radial head fracture. No associated bony injuries could be detected.

**Figure 3:**
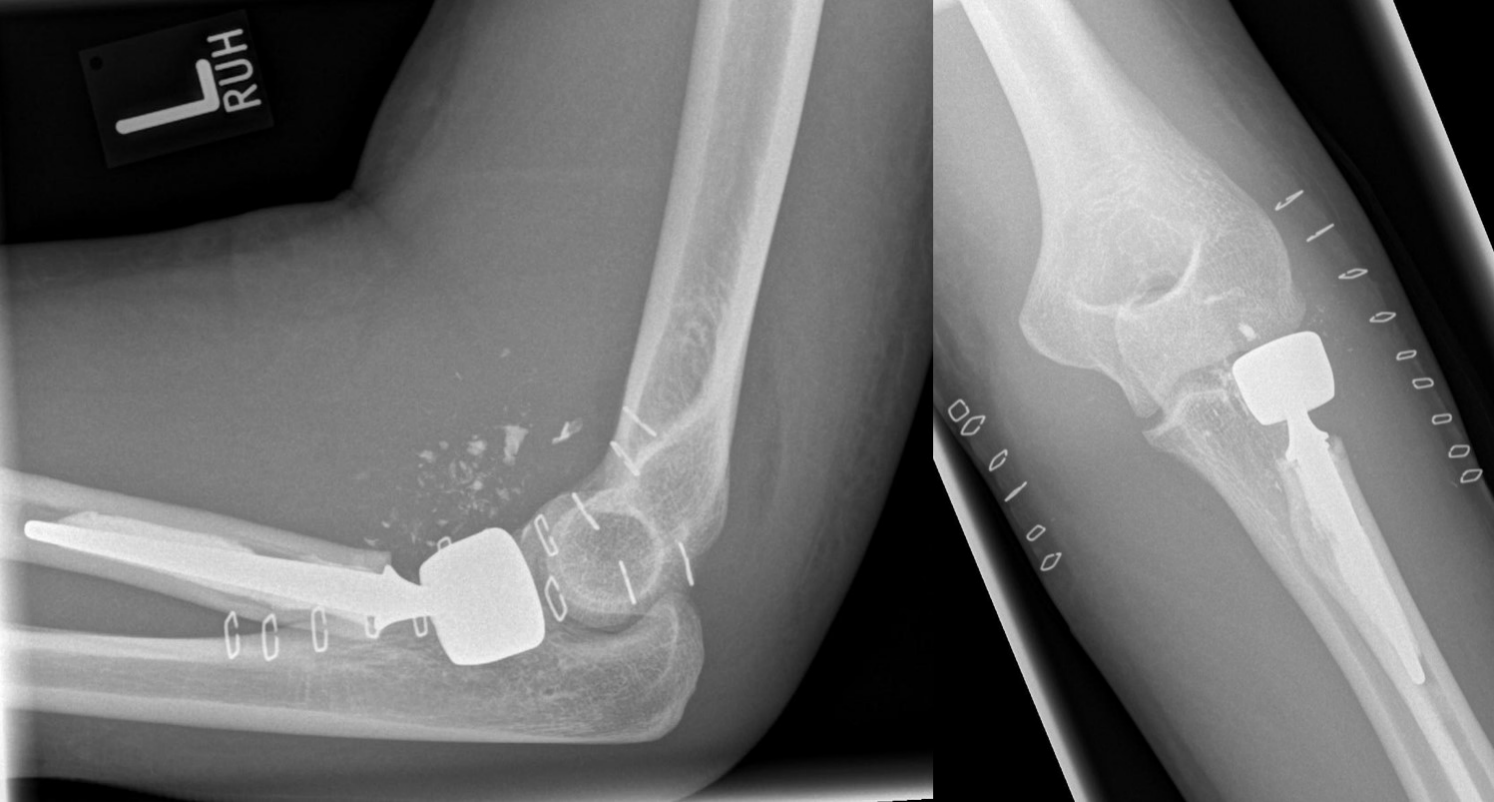
Postoperative radiographs after implantation of a cemented bipolar radial head prosthesis

**Figure 4:**
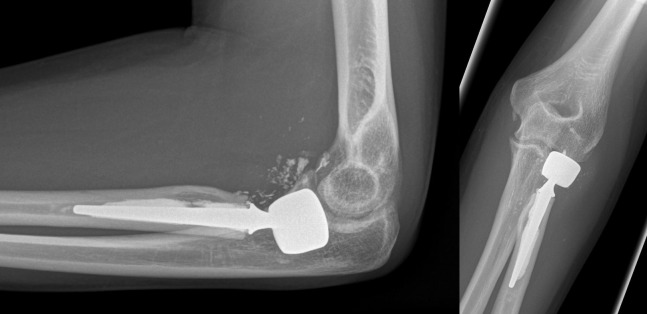
Same patient 12 months after the injury: grade 1 degenerative changes and heterotopic ossifications at the elbow, but good to excellent functional results. No signs of loosening of the radial head prosthesis.

Over a period from 2012–2014 twelve adult patients with an acute Mason type III fracture of the radial head were treated surgically. Preoperatively, all patients received a CT-Scan of the injured elbow joint to detect associated injuries and improve preoperative classification and planning. Radial head fractures were classified according to Mason [7], fractures of the coronoid process according to Regan and Morrey [8], respectively. Demographics, injury pattern, and operative data were extracted from chart review. In all patients the comminuted radial head was resected and a cemented bipolar radial head prosthesis (CRFII, Tornier, Montbonnot, France) was implanted (Figure 1 (Fig. 1), Figure 2 (Fig. 2), Figure 3 (Fig. 3), Figure 4 (Fig. 4)). All prostheses were implanted primarily. Exclusion criteria were presentation more than two weeks after the injury and performance of the radial head arthroplasty as a second-stage or salvage procedure. Furthermore, patients with a Monteggia fracture, Monteggia like lesion/equivalent or Essex-Lopresti injury were excluded from this study. Further exclusion criteria were associated fractures of the distal humerus or the proximal ulna other than coronoid fractures. All of the twelve patients with a unreconstructable Mason Type III radial head fracture could be included in this study. Follow-up examinations were done at an average of 12.7 months after the trauma (range: 11–13 months). All patients were evaluated by the same experienced and blinded examiner. The average age was 48.6 years (range, 18–79 years). There were seven women and five men. The dominant arm was injured in the eight cases and all fractures were closed. The most frequent mechanism of injury was a fall from standing height (n=5), followed by a fall from greater than standing height (n=3), motor vehicle accidents (n=2), and sport accidents (n=2). In two patients, the injury was part of a polytrauma. 

No patient had associated neurologic impairment. Four patients suffered an associated fracture of the coronoid process. According to the Morrey classification there were two type I, and two type II coronoid fractures. All type II coronoid fractures were stabilized with independent lag screws after indirect reduction of the fracture. In four patients the radial head fracture was associated with a posterior elbow dislocation. In two of these patients the MCL (medial collateral ligament) was ruptured, in one patient combined with LCL (lateral collateral ligament) tear. In all cases of ligament injury the MCL and LCL was approached and re-attached using trans-osseous sutures or anchors to the medial or lateral epicondyle, respectively.

In all patients immediate passive motion was initiated by a physiotherapist and continuous passive motion (CPM) without any restriction of movement out of the cast was started on day 2 postoperatively. After one week the cast was removed and unrestricted motion was allowed. Patients with re-attached MCL/LCL received a hinged brace for 6 weeks postoperatively without restriction of elbow flexion and extension. Full weight bearing was allowed at 6 weeks postoperatively. Prophylaxis of heterotopic ossification was performed by administration of indomethacine (75 mg/day) for 3 weeks. During the clinical follow-up evaluation the affected elbow was tested for valgus and varus instability in maximum extension and in 30° of flexion. The pivot-shift test [9] was performed in each patient to evaluate posterolateral rotatory instability, and stability was graded as normal, mild, moderate or severely unstable. Grip strength was measured with a Jamar dynamometer (Fabrication Enterprises Inc., White Plains, New York) with the other hand serving as control. After three consecutive bilateral measurements, the grip strength at the injured side was expressed as a percentage of the control, allowing a correction factor of 1.07 for the dominant over the non-dominant hand [10], [11]. Patient satisfaction was determined by a subjective satisfaction questionnaire according to Jungbluth et al. [10]. Pain at rest and during activity was measured using a visual analogue scale (VAS). The ROM of both wrists, forearms and elbow joints was measured using a standard full-circle goniometer. All patients were assessed using the Mayo Modified Wrist Score (MMWS) [12], the Mayo Elbow Performance Score (MEPS), the functional rating index of Broberg and Morrey [13], and the DASH Score [14]. Radiographs in anteroposterior and lateral view of the elbow joint affected were carried out on all patients at the follow-up examination and reviewed by an experienced and blinded radiologist for capitellar osteopenia, degenerative changes, and heterotopic ossifications. According to Lamas et al. capitellar osteopenia was graded as none, mild, moderate or severe [15]. The degree of degenerative changes was classified according to Broberg and Morrey, as grade 0 (normal joint), grade 1 (slight joint space narrowing and minimum osteophyte formation), grade 2 (moderate joint space narrowing and moderate osteophyte formation), or grade 3 (severe degenerative changes with gross destruction of the joint) [13], [15]. Heterotopic ossification was graded as I, II, III or IV according to Brooker et al. [16]. Furthermore, radiographic signs of loosening of the radial head prosthesis (radiolucent lines, osteolysis, and proximal resorption of the radial head) were evaluated according to Popovic et al. [17]. Fracture union at the coronoid process was defined as bridging bone on anteroposterior and lateral radiographs.

## Results

According to the MMWS with an average score of 88.8 (61–100) good results were obtained (8 excellent, 3 good, 1 fair). An excellent result could be determined for the MEPS with an average of 90.8 points (75–100) (8 excellent, 4 good). The functional rating index of Broberg and Morrey averaged 90.1 (62–100) representing a good result (4 excellent, 6 good, 2 fair). The DASH score showed an average of 17.2 (10.8–55.8). Forearm pronation was measured with a mean value of 86.6° (70° to 90°), supination was 82.1° (45° to 90°). Elbow flexion was from a mean of 14.6° fixed flexion (0° to 30°) to 132.5° (110° to 150°). Palmar flexion of the wrist was registered with a mean of 75.8° (60° to 90°), dorsal extension exhibited a mean of 69.2° (40° to 90°). Taken the corrective factor into account grip strength was a mean of 92.6% (89% to 98%) of the contralateral side. No patient exhibited any wrist or elbow instability. With regard to subjective patient satisfaction, the following results were ascertained using the questionnaire: Eleven out of the twelve patients were satisfied with the results of the operation and stated that in retrospect they would undergo the procedure again. Ten patients reported no relevant restrictions of movement. All patients were able to return to their previous workplace and reached the same level of athletic activities as before the accident. The average VAS value was 1.0 (0 to 2) at rest and 1.4 (0 to 3) during activity. Eight patients neither exhibited indications of capitellar osteopenia nor degenerative changes or heterotopic ossifications at the elbow on conventional radiographs. Three patients showed mild osteopenia, grade 1 degenerative changes, and grade 1 heterotopic ossifications. One patient exhibited grade 2 degenerative changes and grade 2 heterotopic changes. No patient required revision surgery. Fracture union was achieved in all patients. No patient showed signs of loosening of the radial head prosthesis. 

## Discussion

The treatment of comminuted radial head fractures remains challenging and is further complicated if associated injuries are present. In cases of comminuted fractures where anatomic reduction and stable fixation cannot be obtained radial head replacement is warranted [18]. This is supported by the observation that reconstruction of a fracture comprising of more than three fragments yields unsatisfactory results [18]. The radial head is considered an important secondary stabilizer of the elbow [19]. If one of the primary stabilizing structures is injured (fracture of the coronoid, MCL and/or LCL injuries) or if the longitudinal stability of the forearm is compromised (interosseous membrane lesion), the role of the radial head becomes of primary importance [19]. Radial head arthroplasty has proven to restore elbow stability [5]. Therefore, it has superseded radial head resection as the treatment of choice for unreconstructable radial head fractures [5].

Furthermore, radial head fractures are frequently associated with injuries of the collateral ligaments or interosseous membrane [6]. Van Riet et al. demonstrated that the likelyhood of associated injuries correlates with the type of radial head fracture [20]. 75% of patients evaluated with Mason type III fractures had associated injuries. 

A variety of implants have been used to replace the radial head. However, little data exist on the superiority of one design over another. Previously published studies do not allow comparisons between different implants, as comparative studies are not available and indications vary with each type of implant [21].

Most current prosthesis are designed as “unipolar” or “bipolar” devices. Yian et al. have shown that malalignment of unipolar implants can lead to decreased radiocapitellar contact area and increased cartilage wear due to stress concentration [22]. Bipolar radial head implants were developed to maximize radiocapitellar congruency and reduce contact forces [21]. Encouraging short-term results have been demonstrated for unipolar as well as bipolar implants by several authors [1], [4], [5], [17], [19], [20], [23], [24].

Overall, the short-term results following treatment with the bipolar radial head prosthesis evaluated in this case series are promising. Most of our patients showed good or excellent results in the elbow and wrist scores selected, the range of motion as well as the grip strength assessed one year after radial head arthroplasty. Rates of complication and revision surgery can be considered low. No patient exhibited any wrist or elbow instability. Furthermore, most patients exhibited only mild degenerative changes and heterotopic ossifications. Clinically and radiographically, the presented short-term results are similar or better to those reported in the literature. These results suggest that in the short-term, the bipolar radial head prosthesis used in this cohort provides an overall satisfactory clinical outcome with respect to stability, motion, and strength.

Popovic et al. reported mid-term results of 51 patients at a mean of 8.4 years postoperatively. All patients received a cemented Judet’s bipolar radial head prosthesis similar to the implant used in our study [17]. According to the MEPS (83 points on average) 14 patients showed excellent, 25 good, 9 fair, and 3 poor results. However, in 37 (53%) patients radiolucent lines suggesting progressive osteolysis were observed. Progressive loss of bone stock at the radial neck region was observed in 16 (31%). Five (10%) patients had loosening of the prosthesis. Thirty elbows had radiographic evidence of posttraumatic osteoarthritis. According to the classification system of Broberg and Morrey posttraumatic ulnohumeral osteoarthritis was graded as mild in 21 patients, moderate in seven and severe in two. In these patients an increase of pain during daily activities could be attributed to this degenerative arthritis [17]. Twentyone elbows demonstrated heterotopic ossification graded as mild in 13, moderate in six and severe in two patients. In contrast to our study Popovic et al. included severe associated injuries like Monteggia fractures an equivalents. Due to their complexity these injury patterns should be treated and evaluated as separate clinical entities [25]. Furthermore, Monteggia injuries with associated radial head and neck fractures tend to have even poorer outcomes [25], [26], [27], [28]. In order to create a more homogenous and comparable study cohort we intentionally excluded Monteggia injuries and equivalents as well as the even more complex Essex-Lopresti injuries from our study. However, the mid-term results presented by Popovic et al. are overall satisfactory.

Burkhart et al. presented overall promising mid to long-term results after 8.8 years after treatment with a cemented Judet’s bipolar radial head prosthesis similar to the one used in this study [5]. The authors examined 17 patients and according to the MEPS (mean 90.8) six of them achieved excellent results, 10 good, and one fair. The authors presented a mean DASH score of 9.8. Signs of ulnohumeral osteoarthritis were found in 12 patients (eight mild; four moderate), whereas eight patients showed degerative changes of the capitellum (mild to moderate in seven, severe in one patient). However, Burkhart et al. found no evidence of loosening of the implant. Periarticular ossifications could be seen in 13 cases (nine mild, two moderate, two severe). Further complications included two prosthetic dislocations immediately postoperatively due to overstuffing of the humeroradial joint [5]. Comparable to our study mild signs of degenerative changes did not correlate to pain. Similar to Popovic et al. the study cohort was inhomogeneous: six Monteggia like lesion and one Essex-Lopresti injury were included. Furthermore, in contrast to our study, where all prosthesis were implanted primarily, Burkhart et al. reported about seven prosthesis secondarily implanted for trauma, and one for treatment of a chondrosarcoma. The authors noted that the indications for radial head replacement were variable in their study, and a high number of concomitant fractures of the ipsilateral elbow and arm may have influenced the results [5].

Brinkman et al. reported short-term results of 11 patients treated with the cemented Judet’s bipolar radial head prosthesis after an average of 2 years [23]. According to the Elbow Function Assessment Scale and the Modified Andrews Elbow score good or excellent results were achieved in all patients. Radiographically no signs of loosening or hetereotopic ossification could be observed. However, two patients required revision surgery for prosthetic dislocations. Clinically and radiographically our results are comparable to those reported by Brinkman et al. However, these authors implanted all prosthesis secondarily after failed osteosynthesis and/or radial head resection.

In contrast to the aforementioned studies all of our patients received a CT scan preoperatively to evaluate the severity of all existing concomitant injuries of the elbow joint. All radial head and coronoid fractures could be detected and classified. Furthermore, all patients received a standardized postoperative treatment including early immediate continuous motion out of the cast or, in case of collateral ligament injuries, with a hinged brace starting on day 2 after the operation.

## Conclusions

Overall, the good to excellent short-term results that we have recorded look encouraging. Further studies with long-term follow-up and more number of cases will be required to see if these short-results are maintained over time.

## Notes

### Competing interests

The authors declare that they have no competing interests.
